# Cinnamaldehyde and Hyperthermia Co-Treatment Synergistically Induces ROS-Mediated Apoptosis in ACHN Renal Cell Carcinoma Cells

**DOI:** 10.3390/biomedicines8090357

**Published:** 2020-09-17

**Authors:** Chae Ryeong Ahn, Jinbong Park, Jai-Eun Kim, Kwang Seok Ahn, Young Woo Kim, Minjeong Jeong, Hong Jun Kim, Sun Hyang Park, Seung Ho Baek

**Affiliations:** 1 College of Korean Medicine, Dongguk University, 32 Dongguk-ro, Ilsandong-gu, Goyang-si, Gyeonggi-do 10326, Korea; cofud2917@naver.com (C.R.A.); herbqueen@dongguk.ac.kr (J.-E.K.); ywk@dongguk.ac.kr (Y.W.K.); 2 Department of Surgery, Beth Israel Deaconess Medical Center/Harvard Medical School, 330 Brookline Ave, Boston, MA 02215, USA; thejinbong@khu.ac.kr; 3 College of Korean Medicine, Kyung Hee University, 24 Kyungheedae-ro, Dongdaemun-gu, Seoul 02447, Korea; ksahn@khu.ac.kr; 4 College of Korean Medicine, Woosuk University, 443 Samnye-ro, Samnye-eup, Wanju-gun, Jeollabuk-do 55338, Korea; vocation0313@gmail.com (M.J.); kimboncho@woosuk.ac.kr (H.J.K.); 5 Department of Physiology, Research Institute for Endocrine Sciences, Medical School, Jeonbuk National University, 567 Baekje-daero, Dukjin-gu, Jeonju-si, Jeollabuk-do 54896, Korea; beryls@naver.com

**Keywords:** renal cell carcinoma, cinnamaldehyde, hyperthermia treatment, synergism, apoptosis, reactive oxygen species

## Abstract

Renal cell carcinoma (RCC) represents the most common form of kidney cancer, which accounts for 3–5% newly diagnosed cancer cases. Since limited therapies are available for RCC, a search for new options is required. Therefore, in this study, we evaluated the combination effect of cinnamaldehyde (CNM) and hyperthermia treatment. CNM treatment combined with 43 °C hyperthermia synergistically increased cytotoxicity in RCC cell line ACHN cells. Through Western blot assays, we observed increased apoptosis signaling and decreased proliferation/metastasis signaling, along with a repressed heat shock protein 70 level. In flow cytometry analyses, CNM and hyperthermia combination clearly induced apoptosis and mitochondrial potential of ACHN cells, while arresting the cell cycle. Investigation of reactive oxygen species (ROS) suggested a significant increase of ROS generation by CNM and 43 °C hyperthermia co-treatment. We could verify that ROS is crucial in the apoptotic action of combination treatment with CNM and hyperthermia through further experiments regarding an ROS scavenger. Overall, we suggest CNM and hyperthermia combination treatment as an alternative option of anticancer strategies for RCC.

## 1. Introduction

Kidney cancer is the 6th most frequently diagnosed cancer in men and the 10th in women, accounting for 5% and 3% of newly diagnosed cancer cases in 2018 worldwide [1]. Clear cell renal cell carcinoma (RCC) represents the most common form of kidney cancer, which makes up approximately 80% of the total [2]. An increase of RCC incidence has been observed, probably due to improved early detection techniques; however, the 5-year survival rate has increased during the past 30 years. In the USA, 5-year relative survival rates increased from 50% in 1975–1977 to 74% in 2007–2013 [1]. Yet, the overall prognosis remains poor. According to the World Health Organization, more than 14,000 deaths per year result from RCC, ranking as the 13th cause of death from cancer, as approximately 17% of RCCs develop remote metastasis [3].

First-line treatment for RCC varies among risk rate (good, intermediate, and poor risk calculated by the tumor stage, size, grade, and necrosis (SSIGN) score method [4]) of patients, but the standardized guideline suggests a targeted therapy cocktail with tyrosine kinase inhibitors, vascular endothelial growth factor inhibitors, and mammalian target of rapamycin (mTOR) inhibitors [5]. However, these therapies frequently have side effects. Therefore, a novel therapeutic approach for the treatment of RCC is urgently required.

In line, natural products have been gaining interest as one of the potential materials for effective RCC treatment. Cinnamaldehyde (CNM), the main component of the essential oil of cinnamon, is mainly used as a flavoring agent in cinnamon-related foods [6]. It has been proved to exert anticancer effects in various types of cancers, such as colorectal cancer [7], liver cancer [8], and myeloid [9].

Hyperthermia is another candidate approach, which potentially induces apoptosis of cancer cells. Hyperthermal stimuli can induce various physiological responses in cells, such as alteration in the membrane permeability, modification of the cytoskeletal system, changes in macromolecule synthesis, intracellular signal transduction, and inhibition of DNA repair [10]. In RCC, Onishi and colleagues reported the efficacy of hyperthermia on its anticancer effects in vivo [11], and later on, Qi et al. showed the apoptotic feature of hyperthermia treatment using 786-O RCCs [12].

In this study, we assessed the synergistic effect of a combination treatment of CNM and hyperthermia on apoptosis of ACHN cells, a cell line established from metastatic RCC [13], and investigated the associated molecular mechanisms.

## 2. Materials and Methods

### 2.1. Reagents

CNM was purchased from Sigma-Aldrich (St. Louis, MO, USA) and was prepared in dimethyl sulfoxide (DMSO) (Samchun Chem, Seoul, Korea). CNM solutions of 70, 80, and 90 μM were kept at 4 °C until usage.

### 2.2. Cell Culture

The renal adenocarcinoma cell line ACHN cells were obtained from the Korean Cell Line Bank (Seoul, Korea) and maintained in Dulbecco’s Modified Eagle Medium (DMEM) medium supplemented with 10% fetal bovine serum (FBS) (Gibco, Grand Island, NY, USA) and 1% penicillin-strep (Gibco, Grand Island, NY, USA) at 37 °C in an incubator with humidified air with 5% CO₂.

### 2.3. Hyperthermia

ACHN cells were seeded in a 6-well plate at a density of 3 × 10^5^ cells/well, suspended in 3 mL of media, followed by immersion in a temperature-controlled water bath at 37 or 43 °C for 30 min. CNM at the indicated concentrations was added to the samples 1 h prior to hyperthermia treatment.

### 2.4. 3-(4,5-dimethylthiazol-2-yl)-2,5-diphenyltetrazolium bromide (MTT) Assay

The MTT assay was performed to detect cell proliferation following exposure to CNM with hyperthermia. ACHN cells were seeded in 96-well plates (100 μL/well) at a density of 1.5 × 10⁴ cells/mL and allowed to adhere overnight. Then, various concentrations of CNM (70, 80, and 90 μM) were added and the plates were incubated at 37 °C for 1 h in a humidified atmosphere containing 5% CO_2_, followed by immersion in a temperature-controlled water bath at 37 or 43 °C for 30 min. After 48 h of incubation at 37 °C, 5% CO_2_, 20 μL of MTT (2 mg/mL in phosphate-buffered saline (PBS)) (AMRESCO, Solon, OH, USA) were then added to each well and cultured for another 2 h. Following this, the culture media were removed, and the cells were lysed in 100 μL of DMSO. Absorbance was measured with an automated spectrophotometric plate reader at a wavelength of 570 nm. Cell viability was normalized as relative percentages in comparison with untreated controls. The synergy effect of the drug and hyperthermia combinations was determined based on the combination index (CI) calculated by Compusyn software ver. 1.0 (ComboSyn, Inc., Paramus, NJ, USA).

### 2.5. Trypan Blue Assay

Cell viability was determined by a Trypan blue assay by using a hemocytometer after Trypan blue (Sigma-Aldrich, St. Louis, MO, USA) staining (0.4%, 1:1 dilution in the cell containing PBS). Briefly, ACHN cells were seeded in a 6-well plate at a density of 3 × 10^5^ cells and subsequently treated with CNM for 1 h and hyperthermia for 30 min. After 24 h of post-treatment incubation, cells were harvested, diluted by 1:4 in PBS, stained, and counted. The cell survival rate was calculated as the following:

Cell survival rate = Viable cell count/Total cell count × 100%.

### 2.6. Morphology Assay

The cell growth was measured by a morphology assay. ACHN cells were seeded in a 6-well plate at a density of 3 × 10^5^ cells. When the cells had anchored to the plates, they were treated with 70, 80, and 90 μM of CNM for 1 h and incubated at 37 or 43 °C for 30 min. After 24 h, cells were visualized and representative images were captured by a microscope (CX-40, Olympus, Tokyo, Japan).

### 2.7. Wound Healing Assay

The wound healing assay was performed as previously described [14]. Cells were seeded in a 6-well plate at a density of 5 × 10^5^ cells and cultured at 37 °C. When cells grew to confluence, a thin scratch was created in each well with a yellow pipette tip. Images were captured (0 h) by a microscope (CX-40, Olympus, Tokyo, Japan). After 24 h, the culture medium was removed, the cells were washed with PBS, and representative images were captured.

### 2.8. Colony Formation Assay

A total of 400 cells/well were seeded in a 6-well plate and incubated overnight. The following day, the cells were treated with 90 μM CNM for 1 h and consequently incubated at 37 or 43 °C for 30 min for hyperthermia treatment. After 2 weeks, the cells were stained with crystal violet (Sigma-Aldrich, St. Louis, MO, USA) solution at room temperature (RT) for 10 min and washed with PBS. Images of colonies were captured by a microscope (CX-40, Olympus, Tokyo, Japan).

### 2.9. Western Blot Analysis

Western blot analysis was carried out as previously described [15]. Briefly, protein concentrations from isolated ACHN cells were determined. Equal amounts of lysates resolved on sodium dodecyl-polyacrylamide gel electrophoresis (SDS-PAGE) were transferred to a polyvinylidene difluoride (PVDF) membrane, and the membrane was blocked with 1 × TBS containing 0.1% Tween 20 and 5% skim milk at room temperature. After the blocking, the membranes were incubated overnight at 4 °C with the respective primary antibodies of anti-caspase-3, anti-HSP70, anti-caspase-8, anti-caspase-9, Anti-p-ERK (Thr202/Tyr204), anti-ERK, anti-p-p38 (Thr180/Tyr182), anti-p38, anti-p-JNK (Thr183/Tyr185), anti-JNK (from Cell Signaling Technology, Danvers, MA, USA), anti-β-actin, anti-Bcl-2, anti-Bcl-xL, anti-Cyclin D1, anti-VEGF, anti-MMP9 (from Santa Cruz Biotechnology, Inc., Dallas, TX, USA), and anti-cleaved caspase (from GeneTex, Inc., Irvine, CA, USA). The membranes were washed three times and incubated with diluted horseradish peroxidase (HRP)-conjugated anti-rabbit or anti-mouse immunoglobulin G (IgG) (Santa Cruz Biotechnology, Inc., Dallas, TX, USA) secondary antibodies for 1 h at RT. The blots were washed with 1 × TBS-T buffer for 10 min, three times between each step. The immunoblot signals were detected with an enhanced chemiluminescence (ECL) kit (EMD Merck Millipore, Billerica, MA, USA).

### 2.10. Flow Cytometry Assays

To measure the ratio of the apoptosis, a Muse^®^ Annexin V & Dead cell kit (Part Number: MCH100105) (EMD Merck Millipore, Billerica, MA, USA) was used. ACHN cells were seeded in 6-well plates at a density of 3 × 10^5^ cells/well. Then, 24 h after treatments with CNM and hyperthermia, ACHN cells were collected and pellets were subjected to an Annexin V and 7-amino-actinomycin D (7-AAD) staining according to the manufacturer’s instructions. After incubation, the cells were analyzed by using the Muse^®^ Cell Analyzer (EMD Merck Millipore, Billerica, MA, USA).

To verify mitochondrial membrane potential, a MitoPotential assay kit (Part Number: MCH100110) (EMD Merck Millipore, MA, USA) was applied according to the manufacturer’s recommendations. In brief, ACHN cells (3 × 10^5^ cells/well) were incubated with combination treatment for 24 h. Following this, cells were stained with MitoPotential working solution and 7-AAD according to the manufacturer’s instructions. The cells were analyzed by using the Muse^®^ Cell Analyzer (EMD Merck Millipore, Billerica, MA, USA).

For cell cycle analysis, ACHN cells (3 × 10^5^ cells/well) in 6-well plates were exposed to co-treatments for 24 h and the cell cycle phase was analyzed. Cells were then collected, fixed in 70% ice-cold EtOH overnight, washed in PBS, and resuspended in PBS containing 1 mg/mL propidium iodide (PI) and 10 mg/mL RNase A in a dark room for 10 min. The cell cycle was determined using the Muse^®^ Cell Analyzer (EMD Merck Millipore, Billerica, MA, USA).

Reactive oxygen species (ROS) generation was measured using a ROS assay kit (Part Number: MCH100111) (EMD Merck Millipore, Billerica, MA, USA). Twenty-four hours after all treatments, ACHN cells were treated with oxidative stress working solution incubated for 30 min at 37 °C. ROS levels were analyzed using the Muse^®^ Cell Analyzer (EMD Merck Millipore, Billerica, MA, USA). *N*-acetylcysteine (NAC) was treated for 1.5 h prior to combination treatment with CNM and hyperthermia.

### 2.11. Statistical Analysis

All numeric values are represented as the mean ± SD. Statistical significance of the data compared with the untreated control was determined using the Student unpaired *t*-test. * *p* < 0.05, ** *p* < 0.01, and *** *p* < 0.001.

## 3. Results

### 3.1. Combination of CNM and Hyperthermia of 43 °C Synergistically Inhibits Cell Proliferation in RCC Cell Lines

**Figure 1:**
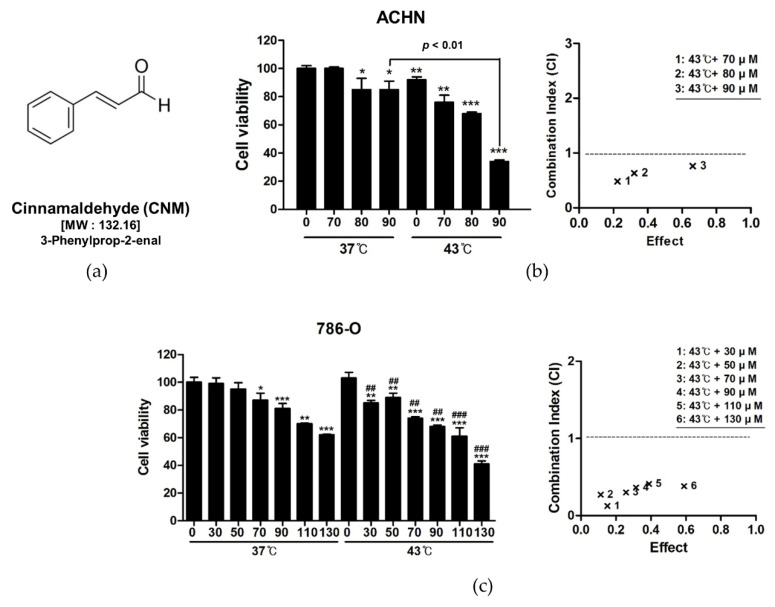
Effect of cinnamaldehyde (CNM) and hyperthermia combination on cell viability in renal cell carcinoma (RCC) cell lines. RCC cell lines, including ACHN and 786-O cells, were treated with CNM with or without hyperthermia of 43 °C and incubated for 24 h. (**a**) Structure of CNM. Cell viability was measured by the 3-(4,5-dimethylthiazol-2-yl)-2,5-diphenyltetrazolium bromide (MTT) assay and the combination index was determined using Compusyn Software in (**b**) ACHN cells and (**c**) 786-O cells. * *p* < 0.05, ** *p* < 0.01, *** *p* < 0.001 vs. 37 °C control group; ^##^ *p* < 0.01, ^###^ *p* < 0.001 vs. hyperthermia treatment group.

First, to verify the anti-proliferative effects of CNM (Figure 1a) and hyperthermia co-treatment, an MTT assay was performed. As shown in Figure 1b, CNM combined with hyperthermia showed a significant decrease in cell viability in RCC cell lines, including ACHN cells and 786-O cells. Moreover, co-treatment with hyperthermia of 43 °C showed dramatically inhibited cell proliferation compared to 37 °C, especially when combined with 90 μM of CNM. Calculation of CI suggested significant synergism when CNM and 43 °C hyperthermia co-treatment was applied. A similar antiproliferative effect and synergistic event by CNM and hyperthermia combination was observed in the 786-O renal cell adenocarcinoma cell line as well (Figure 1c). Further experiments were carried out using ACHN cells since CNM and hyperthermia co-treatment showed a higher inhibition rate in cell viability compared to 786-O cells.

### 3.2. Combination of CNM and Hyperthermia of 43 °C Suppresses Cell Viability, Migration, and Colony Formation of ACHN Cells

**Figure 2:**
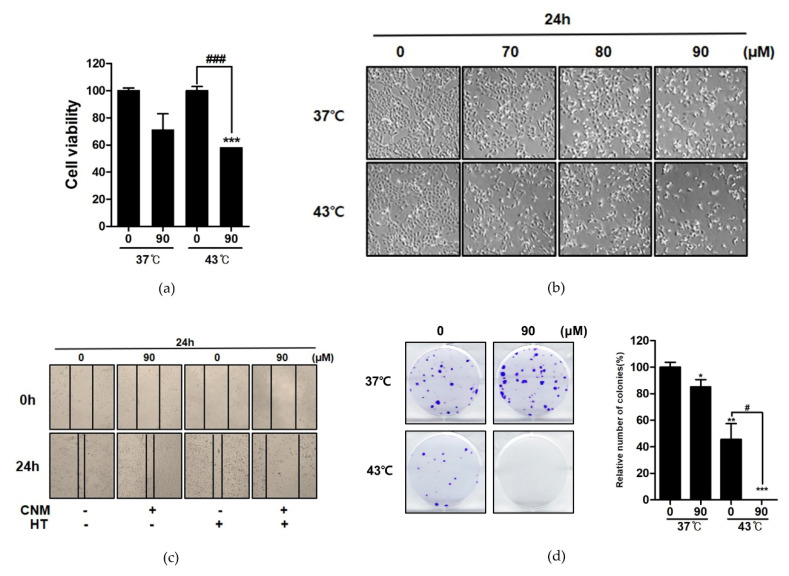
Effect of CNM and hyperthermia combination on cell viability, migration, and colony formation of ACHN cells. ACHN cells were treated with CNM with or without hyperthermia of 43 °C and incubated for 24 h. (**a**) Trypan blue assay was performed and the viable cell portion was determined. (**b**) Morphological changes reflecting apoptosis were visualized and cell viability was counted under a regular light microscope. (**c**) Wound healing assay was performed. (**d**) Clonogenic assay was performed. * *p* < 0.05, ** *p* < 0.01, *** *p* < 0.001 vs. control group; ^#^ *p* < 0.05, ^###^ *p* < 0.001 vs. hyperthermia treatment group.

Trypan blue staining of viable cells (Figure 2a) and visual observation of cell morphology (Figure 2b) confirmed this effect of the CNM and hyperthermia combination. Additional wound healing assays verified the inhibition of cell migration by co-treatment of CNM and hyperthermia (Figure 2c), and furthermore, a dramatic decrease of colony formation was observed in ACHN cells treated with the combination of CNM and 43 °C hyperthermia (Figure 2d).

### 3.3. Combination of CNM and Hyperthermia of 43 °C Increases the Expression of Apoptosis-Associated Factors While Decreasing Protective and Proliferative Factors in ACHN Cells

**Figure 3:**
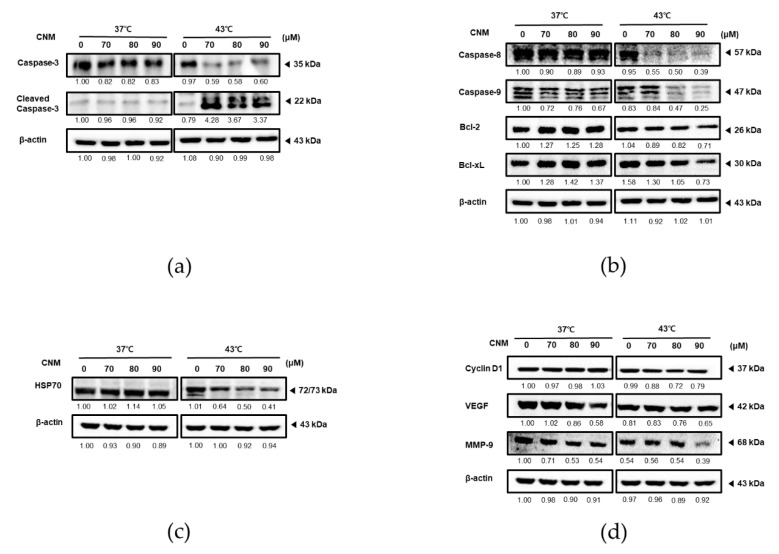
Effect of CNM and hyperthermia combination on the expression of factors of apoptosis, proliferation, survival, and angiogenesis in ACHN cells. ACHN cells were treated with CNM (0, 70, 80, 90 μM) with or without hyperthermia of 43 °C and incubated for 24 h. Whole-cell extracts were prepared, then equal amounts of lysates were analyzed by Western blot analysis. Protein expression of (**a**) caspase-3, (**b**) caspase-8, caspase-9, B-cell lymphoma 2 (Bcl-2), Bcl-xL, (**c**) heat shock protein 70 (HSP70), (**d**) Cyclin D1, vascular endothelial growth factor (VEGF), and matrix metallopeptidase 9 (MMP-9) was measured using Western blot assays. β-actin was used as a loading control. Representative blots are shown.

To elucidate the molecular mechanism participating in the synergistic effect of CNM and hyperthermia co-treatment, we next examined the expression levels of factors related to apoptosis, proliferation, metastasis, and angiogenesis. As in Figure 3a, co-treatment with CNM and hyperthermia of 43 °C greatly induced the cleavage of caspase-3, the final step in programmed apoptosis [16]. However, such an effect was not shown by CNM treatment in 37 °C.

Further proteins in the apoptosis pathway were investigated by additional Western blot assays. The levels of caspase-8 and caspase-9, the main caspases of the extrinsic apoptosis and intrinsic apoptosis, respectively [17], were verified (Figure 3b). In line with the result on cleaved caspase-3, expression of caspase-8 and caspase-9 decreased in a dose-dependent manner; however, this effect was only by the CNM and 43 °C hyperthermia co-treatment. In addition, the anti-apoptotic members of the B-cell lymphoma 2 (Bcl-2) family, Bcl-2 and Bcl-xL [18], were suppressed by the combination treatment of CNM and 43 °C hyperthermia as well.

Next, we assessed the change in HSP70 expression induced by CNM and hyperthermia co-treatment. In response to thermal stress, HSP70 acts to protect cells from protein damage, partial unfolding, and aggregation [19]. To verify whether HSP70 was involved in the action of CNM and hyperthermia, we conducted Western blot assays to find out that HSP70 was suppressed only in the cells co-treated with the CNM and 43 °C hyperthermia combination but not 37 °C (Figure 3c).

Finally, to evaluate the effect of the CNM and hyperthermia co-treatment on the metastatic potential of ACHN cells, the expression levels of Cyclin D1, vascular endothelial growth factor (VEGF), and MMP-9 were measured. Cyclin D1, a regulatory factor of cell adhesion and migration, has been reported to be associated with cancer cell invasion and metastasis [20]. On the other hand, VEGF is the key molecule of angiogenesis [21], and MMP-9 is the most important member of the MMP family, which involves in tumor metastasis [22]. Our results clearly demonstrated these metastatic factors were inhibited by CNM treatment, and the inhibitory effect, especially on MMP-9, was further enhanced when CNM was combined with 43 °C hyperthermia (Figure 3d).

### 3.4. Combination of CNM and Hyperthermia of 43 °C Induces Apoptosis by Arresting Cell Cycle in ACHN Cells

**Figure 4:**
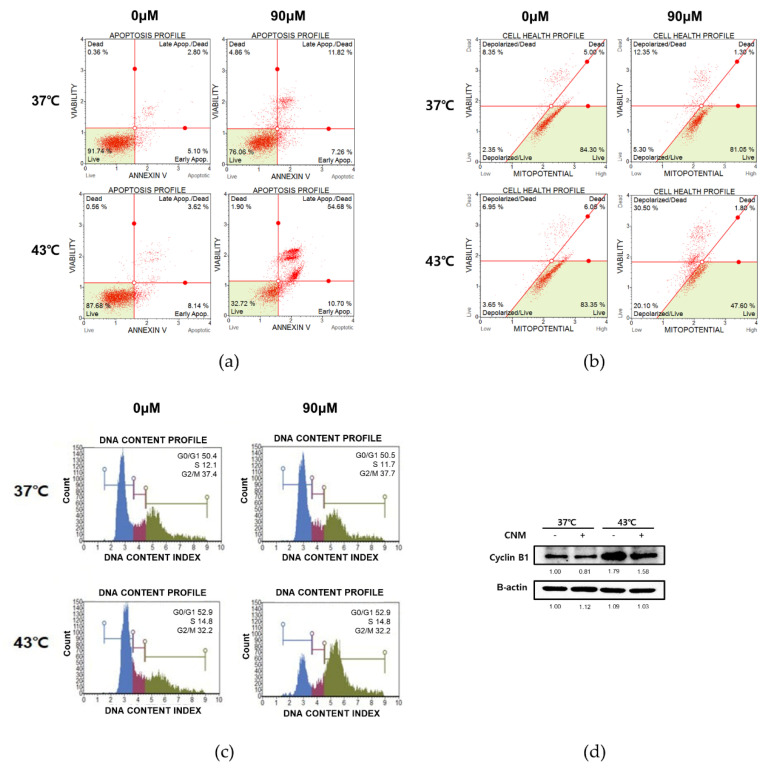
Effect of the CNM and hyperthermia combination on apoptosis, mitochondrial membrane potential, and cell cycle in ACHN cells. ACHN cells were treated with CNM (0, 90 μM) with or without hyperthermia of 43 °C and incubated for 24 h. Flow cytometry analysis on (**a**) apoptosis, (**b**) mitochondrial membrane potential, and (**c**) cell cycle was performed. Green area indicates the live cell portion. (**d**) Cyclin B1 expression was measured by a Western blot assay. Blue area indicates cells in the G0/G1 phase, red area indicates cells in the S phase, and green area indicates cells in the G2/M phase. β-actin was used as a loading control. Representative blots are shown.

Cell cycle arrest is closely related to the induction of apoptosis; thus, it is frequently used as the therapeutic target of anticancer agents [23]. To investigate whether cell cycle arrest also occurs in the action mechanism of CNM and hyperthermia combination treatment, flow cytometry analyses were carried out. As shown in Figure 4a, CNM treatment accompanied by 43 °C hyperthermia clearly increased the Annexin V-associated apoptotic profile of ACHN cells when compared to either sole treatment of 43 °C hyperthermia or the combination of CNM and 37 °C normothermia. Mitochondrial membrane potential changes have been implicated in apoptosis, as depolarization of the inner mitochondrial membrane potential is a reliable indicator of cellular health [24]. Therefore, we analyzed the changes of the mitochondrial membrane potential in ACHN cells. The results suggested that the combination treatment with CNM and 43 °C hyperthermia showed a drastic change in the dead cell count than the 37 °C co-treatment (30.50% vs. 12.35%) consistent with the Annexin V staining results (Figure 4b). Next, we analyzed the profile change of the cell cycle in cells treated with CNM and hyperthermia. As in Figure 4c,d, results indicated cell cycle arrest was induced at the G2/M phase by co-treatment of CNM and 43 °C hyperthermia, and this was due to a decrease in Cyclin B1, the key regulator of cellular mitosis [25].

### 3.5. Combination of CNM and Hyperthermia of 43 °C Increases ROS Signaling in ACHN Cells

**Figure 5:**
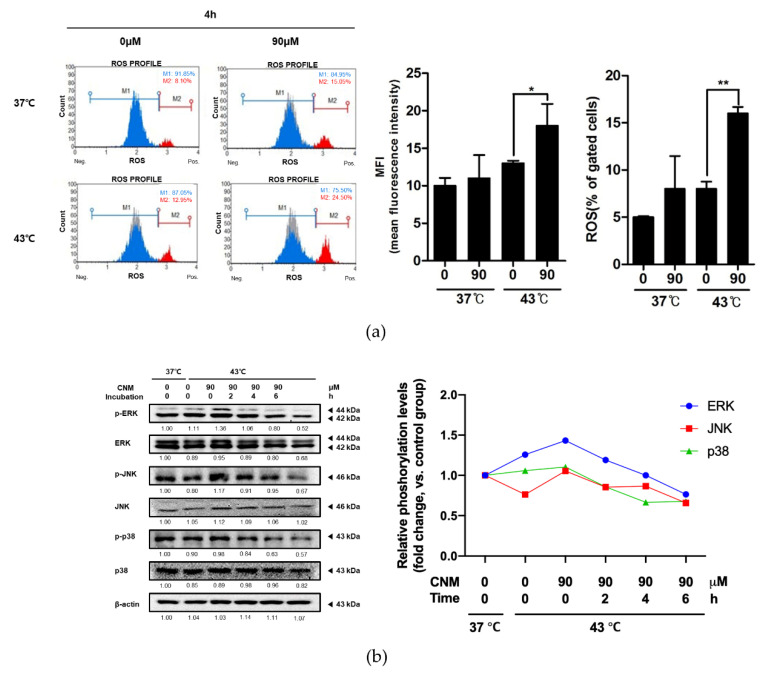
Effect of CNM and hyperthermia combination on ROS generation and MAPK pathway in ACHN cells. ACHN cells were treated with CNM (0, 90 μM) with or without hyperthermia of 43 °C and incubated for 4 h. (**a**) Flow cytometry analysis on ROS generation was performed. Blue area indicates cells in M1 phase, red area indicates cells in M2 phase and gray area indicates cells in 37 °C control group. (**b**) Protein expressions of p-ERK, ERK, p-JNK, JNK, p-p38 and p38 MAPKs were measured using western blot assays. β-actin was used as a loading control. Representative blots are shown. * *p* < 0.05, ** *p* < 0.01.

The molecular mechanism of hyperthermia treatment leading to apoptosis in cancer cells is known to be related to the ROS signaling pathway [10]. Furthermore, ROS itself is already considered as a promising molecular target for the treatment of cancer [26], as well as a novel target for herbal treatments in cancer [27]. Thus, ROS signaling was assessed using a flow cytometry assay kit. As shown in Figure 5a, co-treatment with CNM and 43 °C hyperthermia resulted in a highly increased ROS intensity compared to cells that underwent the 43 °C hyperthermia treatment only. The phosphorylation levels of mitogen-activated protein kinases (MAPKs), crucial regulators of cellular fate [28] but also important downstream pathways of ROS signaling [29], were evaluated by a Western blot assay as well. As a result, CNM and hyperthermia combination treatment induced increase of phosphorylation of all three MAPKs (1.43-fold, 1.05-fold and 1.10-fold vs. 37 °C control group in ERK, JNK and p38, respectively, calculated by phosphor-form/total-form). When compared to 43 °C hyperthermia-treated control ACHN cells, CNM increased phosphorylation of ERK (14.4% increase) and JNK (36.8% increase) but not p38 at the starting point of incubation, by a gradual time-dependent decrease (Figure 5b).

### 3.6. Apoptotic Effect of Combination of CNM and Hyperthermia of 43 °C in ACHN Cells is Dependent on ROS Signaling

**Figure 6:**
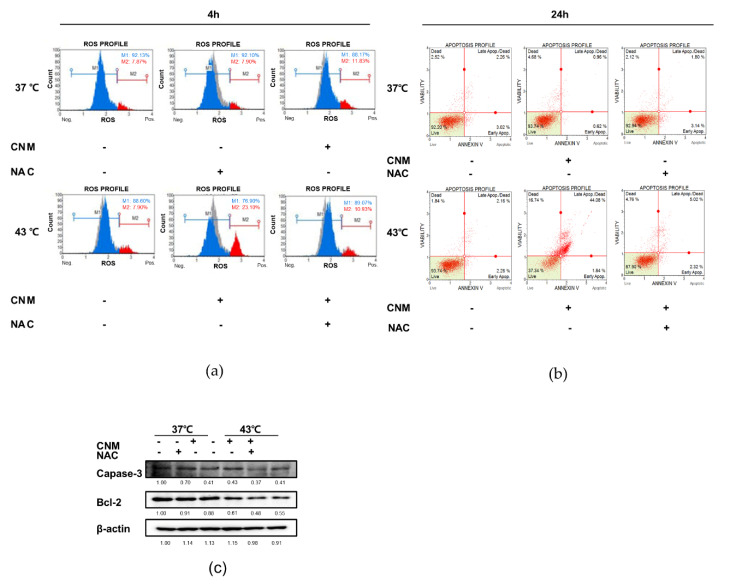
Effect of CNM and hyperthermia combination on ROS generation and apoptosis after *N*-acetylcysteine (NAC) treatment in ACHN cells. ACHN cells were treated with CNM (0, 90 μM) with or without hyperthermia of 43 °C and incubated for 24 h. Flow cytometry analysis on (**a**) ROS generation and (**b**) the apoptosis profile was performed. The blue area indicates cells in the M1 phase, the red area indicates cells in the M2 phase, and the gray area indicates cells in the 37 °C control group. The green area indicates the live cell portion. (**c**) Protein expressions of caspase-3 and Bcl-2 were measured using Western blot assays. β-actin was used as a loading control. Representative blots are shown.

Our next goal was to evaluate the exact role of ROS in the action mechanism of the combination treatment with CNM and hyperthermia. We applied NAC, a scavenger of free radicals and thus an ROS inhibitor [30], prior to the combination treatment of CNM and hyperthermia. NAC pre-treatment indeed suppressed ROS expression (Figure 6a). Moreover, as shown in Figure 6b, further analysis regarding Annexin V indicated that the CNM and hyperthermia treatment failed to induce apoptosis in ACHN cells, suggesting the key role of ROS in the effect of such a combination treatment. In addition, neither the expression of caspase-3 nor Bcl-2 was not altered by co-treatment of CNM and hyperthermia when ROS signaling was inhibited by NAC pre-treatment (Figure 6c).

## 4. Discussion

Kidney cancer is responsible for a worldwide cumulative mortality risk of 0.3% in males and 0.1% in females [3]. Despite improvement in the diagnosis and management of RCC, the most frequently observed form of kidney cancer, it remains one of the most lethal urological malignancies. Thus, the search for effective therapies for RCC care is still an ongoing challenge.

Cinnamon or cinnamomi cortex, the dried bark of *Cinnamomum cassia*, is a widely used herbal medicine in traditional Korean medicine. Originally used to improve blood circulation and tonify kidney Yang Qi in traditional Korean medicine [31], numerous clinical and experimental reports also suggest its beneficial effect in inflammation [32,33] and certain types of cancer. Sadeghi et al. reviewed the role of cinnamon in cancer well, focusing on its apoptotic action [34]. CNM is the organic compound, which composes about 90% of the essential oil of cinnamon [35]. Numerous studies report the anticancer effects of CNM; however, there is as yet no evidence supporting the anticancer effect of CNM in kidney cancer.

As conventional therapy towards cancer has struck a major obstacle, fatal side effects, various approaches have further been made to safely and effectively conquer cancer. Hyperthermia is one of them. Research has shown that hyperthermal treatment in cancer patients can kill cancer cells and shrink tumors by damaging proteins and the structure of them, while causing minimal injury to normal tissues [36]. Although many challenges must be overcome before being considered a standard treatment for cancer, numerous trials have reported the efficacy of hyperthermia treatment as an adjuvant therapy for several types of cancer [37,38,39]. In addition, the combination treatment of hyperthermia with nutritional/herbal supplementations together can potentiate the anticancer effect of each other [40,41]. Thus, in this study, we attempted to verify the combination effect of hyperthermia and CNM in the ACHN RCC cell line. By observation of the cell viability, morphology, and migration, we can conclude that the combination therapy of CNM and hyperthermia synergistically inhibited the proliferation of ACHN cells (Figure 1 and Figure 2).

The apoptosis of cells can be subcategorized into intrinsic and extrinsic pathways. Either triggered by death receptors (extrinsic apoptosis) or sensed within the cell itself (intrinsic apoptosis), downstream heads towards cleavage of caspase-3, the final step of programmed cell death [16]. Our results showed that the combination treatment with CNM and hyperthermia of 43 °C greatly induced the cleavage of caspase-3 (Figure 3a), implying the induction of apoptosis.

The intrinsic pathway of apoptosis involves the response of mitochondria. During apoptosis, cytochrome c is released from mitochondria and binds with apoptotic protease-activating factor-1 and ATP, and then binds to pro-caspase-9 to create an apoptosome complex, which cleaves caspase-9 and in turn activates caspase-3 [17]. On the other hand, extrinsic apoptosis is initiated by the tumor necrosis factor receptor (TNFR) family. Activated TNFRs result in the proteolytic cleavage of caspase-8, inducing cleavage of caspase-3. Moreover, following activation of TNFRs, the balance between pro-apoptotic and anti-apoptotic members of the Bcl-2 family shifts to an outnumbered proportion of anti-apoptotic members, such as Bcl-xL and Bcl-2, leading to the initiation of intrinsic apoptosis as well [18]. Treatment with CNM and hyperthermia together clearly increased levels of cleaved caspase-8, cleaved caspase-9, Bcl-xL, and Bcl-2, indicating the fact that this combination therapy initiates both intrinsic and extrinsic pathways of apoptosis in ACHN cells (Figure 3b).

The cell cycle of eukaryotic cells consists of four phases: G1, S, G2, and M phase. Activation of each phase depends on the proper progression/ completion of the previous phase. After DNA replication in the S phase, cells during the G2 phase continue to grow to enter the mitotic M phase, in which cells divide into two daughter cells. However, cancer is a product of disturbed apoptosis in injured or mutated cells caused by accumulating mutations from an abnormal cell cycle [42]. Our results demonstrated that the combination therapy with CNM and hyperthermia induced cell cycle arrest and thus apoptosis in ACHN cells (Figure 4). Moreover, the metastatic potential was suppressed by the combination treatment as well. The results indicated a decrease in metastatic factors, such as Cyclin D1 [20], VEGF [21], and MMP-9 [22], all suggesting the beneficial effect of CNM and hyperthermia co-treatment in the improvement of metastasis.

HSP70 is known to induce oncogenesis, proliferation, migration, and metastasis while suppressing apoptosis specifically in cancer. Moreover, these proteins allow cancer cells to develop drug resistance to several therapeutic agents, including cisplatin, oxaliplatin and bortezomib [43]. In our results, co-treatment of CNM with 43 °C hyperthermia showed inhibition in HSP70 expression whilst combination with 37 °C normothermia did not, explaining the reason why the combination with 43 °C hyperthermia showed a much higher apoptotic potential than in the 37 °C environment (Figure 3c).

ROS is considered as a crucial pathway within the hyperthermia treatment process. Evidence from hyperthermic intraperitoneal chemotherapy, a treatment in which infusion and circulation of chemotherapy is applied after heating the anticancer drugs, especially in abdominal cancers, shows a definite involvement of ROS in this therapy [44]. Moreover, ROS plays a crucial role in the effect of the combination of hyperthermia with natural products [45,46]. In line with this, our results demonstrated that co-treatment of CNM with 43 °C hyperthermia can be a novel therapeutic approach towards RCC. We observed that by combination with CNM and hyperthermia, ROS signaling was induced in ACHN cells (Figure 5), and ROS was necessary for the apoptotic effect of such a combination treatment (Figure 6). Regarding the previous evidence showing ROS signaling is a potential therapeutic target for RCC treatment [47,48], our results may provide essential evidence for selecting ROS-targeted combination therapy of CNM and hyperthermia.
